# The Gut–Liver IgA Axis in Liver Disease: From Mucosal Immunity to Hepatic Inflammation and Cancer

**DOI:** 10.3390/cells15211933

**Published:** 2026-10-24

**Authors:** Sung Woo Cho, Gil Won Lee, Pil Soo Sung

**Affiliations:** 1 The Catholic University Liver Research Center, Department of Medical Sciences, College of Medicine, The Catholic University of Korea, Seoul 06591, Republic of Korea; 1102csw@catholic.ac.kr (S.W.C.); lgw0429@catholic.ac.kr (G.W.L.); 2 Division of Gastroenterology and Hepatology, Department of Internal Medicine, College of Medicine, Seoul St. Mary’s Hospital, The Catholic University of Korea, Seoul 06591, Republic of Korea

**Keywords:** immunoglobulin A, gut–liver axis, mucosal immunity, alcohol-associated liver disease, metabolic dysfunction-associated steatotic liver disease, hepatocellular carcinoma, liver fibrosis

## Abstract

**Highlights:**

**What are the main findings?**

• IgA supports mucosal protection and contributes to hepatic inflammation, fibrosis, and tumor immune regulation through cellular and antibody-mediated mechanisms.
• Gut–liver IgA pathways differ across disease models; human associations establish neither a shared intestinal origin nor causality.

**What are the implications of the main findings?**

• Future IgA-directed strategies require evaluation of compartment specificity and mucosal safety.
• Clinical translation requires distinguishing prognostic from treatment-selection biomarkers and demonstrating liver benefit beyond IgA changes.

**Abstract:**

Immunoglobulin A (IgA) connects mucosal defense with hepatic immune regulation, but its effects vary by anatomical compartment, antibody properties, and responding cell type. This narrative review synthesizes experimental and clinical evidence on the gut–liver IgA axis in alcohol-associated liver disease, metabolic dysfunction-associated steatotic liver disease and steatohepatitis, and hepatocellular carcinoma. We distinguish IgA-producing cells from secreted antibodies and IgA-responsive cells and examine antibody production, cellular trafficking, hepatic clearance, and biliary export. Experimental studies demonstrate the protective effects of mucosal IgA delivery and the context-dependent contributions of IgA-associated responses to inflammation, fibrosis, and impaired antitumor immunity. Human studies identify tissue and prognostic associations but do not establish a common intestinal source of pathogenic hepatic IgA. Collectively, these findings provide a rationale for investigating disease-specific, compartment-directed approaches targeting antibody production, cellular trafficking, protective export, or local effector responses. Clinical translation requires validation of causal mechanisms and intervention-specific biomarkers, as well as evidence of improved liver outcomes while preserving mucosal protection.

## 1. Introduction

The gut and liver exchange microbial, metabolic, cellular, and antibody signals that regulate hepatic immunity [1,2]. Immunoglobulin A (IgA) is central to this communication: it supports microbial containment at mucosal surfaces and participates in immune responses beyond the intestine [3,4]. Bacterial aggregation exemplifies this barrier function; for example, IgA supplementation reduced bacterial penetration of the mucus layer in ex vivo experiments [5].

The effects of IgA depend on its anatomical location and the responding cell population. Experimental studies link hepatic IgA secretion to protection against alcohol-induced injury while also implicating IgA-associated responses in fibrosis, hepatocyte injury, and tumor immune suppression [2,3,6,7,8]. Human studies also associate serum IgA and IgA-enriched hepatic tissue features with the progression of metabolic dysfunction-associated steatotic liver disease (MASLD) [9]. These observations warrant investigation of whether pathogenic IgA responses can be reduced without compromising mucosal defense.

Addressing this question requires distinguishing the actions of IgA-producing cells from those of the antibodies they secrete and separating cellular trafficking from antibody secretion, transport, and clearance [3,8,10]. We examine these processes in alcohol-associated liver disease (ALD), MASLD and metabolic dysfunction-associated steatohepatitis (MASH), and hepatocellular carcinoma (HCC). We focus on evidence linking individual processes to disease without assuming that they form a single continuous pathway.

Therapeutic opportunities may lie in selectively modulating pathogenic hepatic IgA responses while preserving mucosal protection. We organize these opportunities into four distinct compartments: (1) antibody generation and plasma-cell maintenance, (2) IgA-related cellular trafficking, (3) protective pIgR-dependent export, and (4) local hepatic effector responses. The disease-specific sections that follow compare evidence across these compartments, distinguishing causal interventions in mice, functional assays using human cells, and observational associations in patients. This framework does not assume that increased intestinal IgA production initiates every hepatic IgA-associated phenotype.

### Literature Search and Evidence Selection

This narrative review integrates foundational mechanistic studies and recent experimental and clinical evidence concerning the gut–liver IgA axis. Targeted PubMed searches were conducted from 7 September to 11 October 2026; the final search was performed on 11 October 2026. Search terms included combinations of “immunoglobulin A” or “IgA” with “gut–liver axis”, “alcohol-associated liver disease”, “alcoholic hepatitis”, “MASLD”, “MASH”, “NAFLD”, “NASH”, “hepatocellular carcinoma”, “pIgR”, “B cells”, “fibrosis”, and “Fc receptor”. Additional relevant publications were identified by screening the reference lists of key original studies and reviews.

Studies were selected for their relevance to intestinal IgA generation and trafficking, hepatic IgA uptake and biliary export, disease-associated immune responses, therapeutic interventions, and clinical biomarkers. Original mechanistic studies, experimental animal models, human cell functional assays, human tissue analyses, observational cohorts, and relevant clinical trials were considered. No fixed publication-year restriction was imposed, allowing foundational mechanistic studies to be included. Studies outside the IgA-focused scope and without relevance to the necessary liver-disease context were excluded. Broader liver-disease studies were retained only when needed to contextualize the IgA-focused literature and were not treated as direct evidence for IgA-specific pathways. For mechanistic interpretation, priority was given to original experiments that directly evaluated IgA production, trafficking, receptor-dependent responses, or export; human observational studies were considered separately for clinical relevance. Foundational studies were retained for mechanisms established before the recent disease-focused literature, whereas more recent studies received greater emphasis when evaluating disease-specific findings and translational readiness. These were qualitative selection principles rather than a numerical weighting or formal systematic screening scheme.

Evidence was assessed based on study design, mechanistic specificity, and relevance to human disease. Mouse genetic and interventional studies, human cell functional assays, and clinical observational studies were distinguished to avoid treating associations as evidence of causality. As this was a narrative rather than a systematic review, no formal risk-of-bias assessment or quantitative meta-analysis was undertaken. A narrative approach was chosen to compare compartment-specific mechanisms and identify inferential gaps across heterogeneous evidence, rather than to answer a single predefined intervention–outcome question. Differences in species, anatomical compartments, antibody forms, interventions, and outcomes further precluded a meaningful pooled effect estimate. Because literature identification and study selection were targeted rather than exhaustive, relevant studies may have been missed, and selection bias cannot be excluded.

## 2. Physiological Framework for Bidirectional Gut–Liver IgA Communication

### 2.1. Intestinal IgA Generation and Immune Programming

Intestinal IgA responses develop in gut-associated lymphoid tissues through T-cell-dependent and T-cell-independent pathways. Transforming growth factor-β (TGF-β) promotes IgA induction, while epithelial sensing of bacteria induces secretion of a proliferation-inducing ligand (APRIL), which supports T-cell-independent IgA2 class switching in human intestinal tissue [11,12]. Interactions between B cells and subepithelial dendritic cells in Peyer’s patches are required for IgA production [13], and microbial short-chain fatty acids enhance B-cell metabolism and antibody responses in experimental systems [14].

Dendritic cell-derived retinoic acid promotes gut homing and IgA secretion [15]. Lamina propria plasma cells produce polymeric IgA, which is transported across the epithelium by the polymeric immunoglobulin receptor (pIgR) and released into the lumen as secretory IgA [16]. Secretory IgA supports microbial containment, including by enchaining dividing bacteria [17]. Human intestinal plasma-cell subsets can persist for years or decades, sustaining mucosal antibody production [18].

### 2.2. Connecting Intestinal and Hepatic IgA Compartments

Intestinal and hepatic IgA compartments may communicate through antibody distribution, cell trafficking, and exposure to intestinal antigens, but their relative contributions and directionality have not been established in humans. Moro-Sibilot et al. identified IgA-secreting cells in mouse and human livers; in mice, they traced hepatic IgA-producing plasmablasts to Peyer’s patches and demonstrated reactivity against commensal bacterial antigens [10]. The human observations establish the presence of these cells, whereas the evidence for their cellular origin comes from mouse experiments.

Long-lived IgA plasma cells also occupy intestinal and bone marrow niches in mice, with shared clonality but limited ongoing recirculation [19]. Thus, intestinal priming and subsequent antibody production in an extraintestinal niche are distinct events. For hepatic populations, key questions are where these cells are initially activated and how they persist after recruitment to the liver. In patients with celiac disease, shared antibody sequences linked intestinal plasma cells to serum IgA despite differences in antibody molecular composition between compartments [20]. This demonstrates clonal relatedness but not direct export of intestinally secreted antibody. Moreover, commensal-induced systemic IgA protected mice against polymicrobial sepsis, showing that extraintestinal IgA can contribute to host defense [21]. Hepatic exposure to a mucosally related antibody pool is therefore not inherently pathological.

In the tested ethanol-fed mouse models, impaired pIgR-dependent defense increased bacterial translocation [3]. Separately, mouse tracing experiments identified hepatic IgA-secreting cells with commensal-antigen reactivity [10]. These findings concern distinct experimental processes and do not establish that intestinal barrier dysfunction directly causes hepatic IgA-cell accumulation or a continuous gut-to-liver IgA pathway in humans.

### 2.3. Hepatic IgA Uptake, Clearance, Transport, and Liver-to-Gut Feedback

The liver contributes to IgA homeostasis through uptake, clearance, local secretion, and transport into bile. These processes have distinct consequences: clearance of antibody from the blood does not necessarily imply delivery of intact, functional IgA to the intestine. Studies in mice using recombinant human IgA showed that N-glycan-dependent recognition by the hepatic asialoglycoprotein receptor contributes to clearance, with substantial differences between IgA1 and IgA2 [22]. Molecular composition therefore influences both circulating persistence and tissue exposure.

Early human observations suggested that impaired hepatic IgA clearance or biliary reflux could contribute to increased circulating polymeric IgA [23]. However, these observations neither quantify functional biliary IgA export nor establish increased IgA synthesis.

An early epithelial transport study showed that dimeric IgA could carry bound antigen across pIgR-transfected cells [24]. This demonstrated an in vitro mechanism for immune-complex export; however, its contribution to human biliary clearance has not been quantified.

In early human studies, Nagura et al. localized IgA-producing plasma cells near bile ducts and described ultrastructural findings consistent with secretory component-mediated transport [25]. More recent functional evidence comes from mouse studies: Hendrikx et al. restored hepatocyte pIgR expression in pIgR-deficient animals, thereby increasing intestinal IgA, reducing bacterial translocation, and ameliorating ethanol-induced injury [3]. Thus, the human observations and mouse intervention support complementary aspects of biliary immunity, although the quantitative contribution of this route in humans remains unresolved.

Because production, uptake, and export can change independently during liver disease, serum IgA and luminal antibody delivery should be assessed separately [3,16]. Hepatic pigment epithelium-derived factor (PEDF) is an IgA-independent example of liver-to-gut feedback, which restrains intestinal stem-cell proliferation through Wnt/β-catenin inhibition [26].

### 2.4. Determinants of Protective and Pathogenic IgA Responses

The biological consequences of IgA depend on its anatomical location, molecular organization, antigen recognition, and the receptors and accompanying signals encountered by responding cells. Human serum IgA is predominantly monomeric, whereas mucosal secretory IgA is mainly dimeric and associated with J chain and secretory component [16]. Secretory IgA-mediated bacterial containment therefore has effects distinct from those of tissue immune complexes.

Antigen specificity and microbial context also affect how IgA responses are interpreted. Polyreactive intestinal IgA participates in mucosal homeostasis, whereas IgA coating has been used to identify bacterial taxa that transfer colitis susceptibility in experimental systems [27,28]. These findings support assessing pathogenic responses by function and location rather than IgA positivity alone, within the broader microbial and metabolic gut–liver network [29].

In human myeloid cells, Fcα receptor I (FcαRI; CD89) illustrates how the mode of antibody engagement affects signaling: non-cross-linking ligation can induce inhibitory signaling, whereas receptor cross-linking can trigger activation [30]. Serum IgA immune complexes cooperate with Toll-like receptor stimulation to enhance inflammatory cytokine production in human monocytes, macrophages, and Kupffer cells [31].

Species differences constrain interpretation of these pathways. Standard laboratory mice lack an ortholog of human FcαRI/CD89 [32]; thus, FcRγ-dependent murine phenotypes are not direct evidence of CD89-mediated IgA signaling.

In functional assays using human monocyte-derived macrophages, coated IgA complexes increased programmed death-ligand 1 (PD-L1) expression, an effect prevented by CD89 blockade [8]. By contrast, experimental inflammation-associated hepatocarcinogenesis identified IgA-producing cells that suppressed CD8+ T-cell responses through PD-L1 and interleukin-10 (IL-10) [33]. This cell-mediated mechanism should not be equated with a direct effect of secreted IgA.

**Figure 1:**
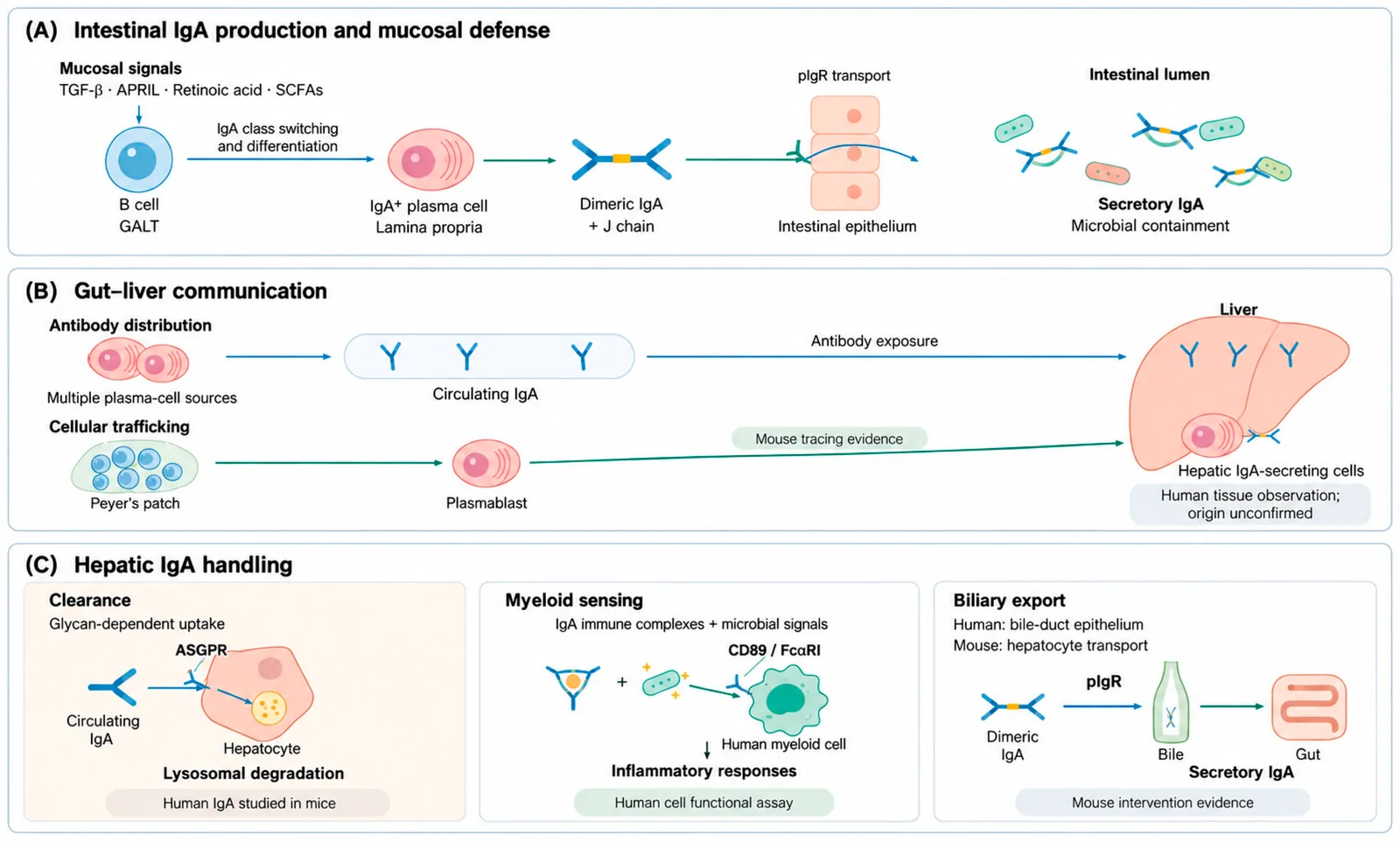
Intestinal IgA production, gut–liver communication, and hepatic IgA handling. (**A**) Intestinal IgA production and mucosal defense. Mucosal signals support IgA class switching and B-cell differentiation in gut-associated lymphoid tissue. Lamina propria plasma cells produce J-chain-containing dimeric IgA, which undergoes pIgR-mediated transport across the intestinal epithelium and is released into the lumen as secretory IgA, supporting microbial containment [11,12,13,14,15,16,17]. (**B**) Gut–liver communication. Antibody distribution and cellular trafficking connect intestinal and hepatic IgA compartments through distinct routes. Circulating IgA derives from multiple intestinal and extraintestinal plasma-cell sources and contributes to hepatic antibody exposure [19,20,21]. Mouse tracing studies demonstrate recruitment of Peyer’s patch-derived plasmablasts to the liver. Although IgA-secreting cells are also present in human liver, their intestinal origin has not been directly established [10]. (**C**) Hepatic IgA handling. Clearance, myeloid sensing, and biliary export are depicted as separate processes. ASGPR-mediated uptake contributes to glycan-dependent IgA clearance and lysosomal degradation; this clearance mechanism was characterized using recombinant human IgA in mice [22]. In human myeloid cells, IgA immune complexes cooperate with microbial signals through FcαRI/CD89 to enhance inflammatory responses [31]. Biliary export provides a route for secretory IgA delivery to the intestine. Early human observations implicate bile-duct epithelium in IgA transport [25], whereas functional evidence for protective hepatocyte pIgR-mediated export derives from mouse experiments: restoring hepatocyte pIgR in ethanol-fed pIgR-deficient mice increased intestinal IgA and reduced bacterial translocation and liver injury [3]. The quantitative contribution of the hepatocyte route in humans remains unresolved. Take-home message: Distinct routes of IgA production, trafficking, clearance, sensing, and export should not be interpreted as a single proven gut-to-liver pathway in humans.

For clarity, we use “IgA-producing” for antibody-secreting cells, “IgA-bound” for cells carrying surface-bound antibody, and “IgA-responsive” only when a functional response has been demonstrated. IgA positivity in tissue sections alone is described as staining or deposition. Antibody form, antigen specificity, immune-complex formation, and experimental presentation should be considered when interpreting IgA function. Figure 1 summarizes the physiological framework.

## 3. The Gut–Liver IgA Axis in Alcohol-Associated Liver Disease

### 3.1. Alcohol-Associated Disruption of Intestinal Immunity

Alcohol-associated liver disease (ALD) develops in an altered intestinal environment in which microbial dysbiosis and barrier dysfunction increase hepatic exposure to microbial products. Experimental transfer of intestinal microbiota from patients with alcohol-associated hepatitis into mice showed that microbial community composition can influence susceptibility to alcohol-induced liver injury [34].

Clinical permeability studies in alcohol dependence identified marked heterogeneity: increased permeability and associated dysbiosis were confined to a subset of patients [35]. A separate microbial mechanism involves cytolysin. Cytolysin-producing *Enterococcus faecalis* was linked to severe disease, and targeted bacteriophages reduced injury in mice colonized with patient-derived microbiota [36]. These studies establish microbial injury pathways without demonstrating that IgA mediates them.

**Figure 2:**
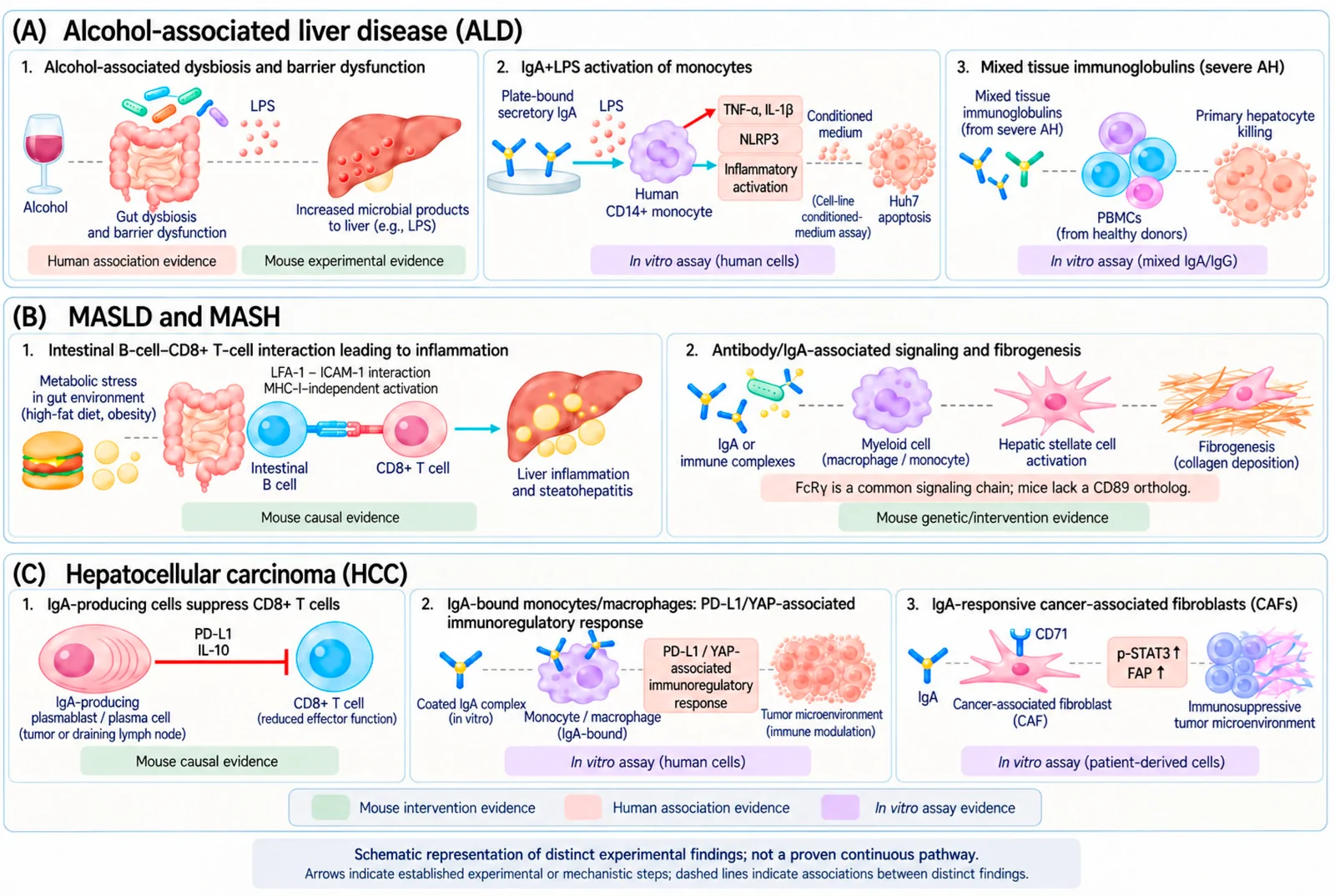
Disease-specific IgA-associated responses. (**A**) Alcohol-associated dysbiosis and barrier dysfunction can increase hepatic microbial exposure; permeability varies among patients, and IgA mediation is not established [34,35]. Plate-bound secretory IgA with LPS enhanced inflammatory responses in human CD14+ monocytes, including increased NLRP3 expression and caspase-1/gasdermin D cleavage. The separate apoptosis branch represents the Huh7-cell conditioned-medium assay, not primary hepatocytes; the responsible mediators remain unresolved [37]. Separately, mixed tissue-extracted immunoglobulins from severe alcohol-associated hepatitis enhanced PBMC-dependent primary human hepatocyte killing; this is not an isolated IgA effect [7]. (**B**) Mouse MASLD/MASH experiments distinguished intestinal B-cell–CD8+ T-cell interactions associated with inflammation from antibody- and FcRγ-dependent fibrosis [2]. FcRγ is a common signaling chain, not an identified IgA-binding receptor; standard laboratory mice lack an ortholog of human FcαRI/CD89. (**C**) HCC mechanisms include PD-L1- and IL-10-dependent suppression by mouse IgA-producing cells [33], CD89/YAP-associated immunoregulatory responses demonstrated in human macrophage functional assays [8], and CD71/STAT3-associated responses in patient-derived CAFs [6]. These represent distinct experimental findings rather than an established continuous causal circuit. Color-coded labels distinguish mouse experimental/interventional evidence (light green), human observational associations (light pink), and in vitro functional assays (light purple). Solid arrows indicate experimentally supported mechanistic steps, whereas gray dashed, non-directional connectors represent conceptual associations between distinct findings without establishing causal continuity. Take-home message Across ALD, MASLD/MASH, and HCC, IgA-associated mechanisms are supported by different experimental designs and must not be merged into a single established causal circuit.

IgA responses in ALD should be considered across distinct compartments. Moro-Sibilot et al. reported increased numbers of hepatic IgA-secreting cells and sinusoidal IgA deposition in experimental ethanol injury, extending their evidence for a Peyer’s patch contribution to hepatic IgA responses [10]. However, hepatic accumulation does not necessarily imply effective intestinal protection. In the tested ethanol-fed pIgR-deficient mouse model, hepatocyte pIgR restoration increased protective intestinal IgA delivery and reduced injury [3]. These intervention data do not establish that IgA retention and impaired luminal export form a single causal sequence in patients. Figure 2A summarizes these distinct ALD pathways.

### 3.2. IgA-Associated Myeloid Inflammation and Hepatocyte Injury

In our published ALD cohort, serum IgA correlated with the hepatic IgA-stained area and CD68-normalized *IL1B* expression [37]. These observations associate IgA with an IL-1β-rich hepatic environment and prompted functional testing of the myeloid response described in Section 2.4. The correlations do not establish the site of antibody production, the direction of any intestinal–hepatic relationship, or a causal role for IgA deposition in ALD.

In functional assays using human cells, plate-bound secretory IgA enhanced tumor necrosis factor-α (TNF-α) and IL-1β production in lipopolysaccharide (LPS)-stimulated CD14+ monocytes and increased NLRP3 expression and cleavage of caspase-1 and gasdermin D [37]. Conditioned medium from these monocytes increased Huh7-cell apoptosis relative to medium from monocytes stimulated with LPS alone [37].

These cell-based findings are consistent with an IgA-amplified inflammatory myeloid response. Separate experimental studies also implicate IL-1 signaling in alcohol-associated steatohepatitis [38]. However, the mediators responsible for the additional Huh7-cell apoptosis remain unresolved. Moreover, immobilized secretory IgA with LPS co-stimulation should not be assumed to reproduce circulating monomeric IgA or native hepatic immune complexes.

### 3.3. Tissue Antibody Responses and Experimental Modulation

In severe alcohol-associated hepatitis, liver-derived IgA and IgG recognized bacterial and host antigens, and mixed tissue immunoglobulins enhanced peripheral blood mononuclear cell (PBMC)-dependent hepatocyte killing [7]. These findings support pathogenic tissue-associated antibody responses but establish neither their intestinal origin nor an IgA-specific cytotoxic effect.

Experimental interventions further distinguish antibody distribution from protective export. In ethanol-fed mice, FTY720 reduced hepatic IgA-associated measures, inflammatory responses, and liver injury; however, its broad effects on lymphocytes preclude an IgA-selective interpretation of trafficking [37]. Conversely, restoring hepatocyte pIgR in deficient mice increased intestinal IgA delivery and reduced injury [3]. Lifelong IgA deficiency did not reduce ethanol-induced injury and was accompanied by increased IgM, suggesting compensation [39]. Together, these studies support context-dependent effects of IgA and caution against treating systemic deficiency, altered trafficking, and restored export as equivalent interventions. Figure 2A distinguishes human myeloid functional testing from mixed-tissue-immunoglobulin experiments.

## 4. The Gut–Liver IgA Axis in MASLD and MASH

### 4.1. Metabolic Programming of Intestinal B Cells

Metabolically activated intestinal B cells promoted CD8+ T-cell activation through LFA-1–ICAM-1 interactions independently of MHC class I-dependent antigen presentation. Activation and steatohepatitis persisted in germ-free mice, while photoconvertible Kaede reporter tracing demonstrated trafficking of intestinal B and T cells to the liver [2]. Other models implicate microbiota-driven hepatic B-cell activation and responses to oxidative stress-derived antigens, indicating that distinct signals can drive B-cell-mediated inflammation [40,41].

In the metabolic model, μMT mice exhibited severely impaired systemic B-cell development because of the absence of membrane-bound IgM but retained intestinal IgA-expressing B cells, with evidence of local intestinal IgA secretion [2]. Under the dietary conditions tested, these mice developed steatohepatitis without detectable fibrosis despite defective peripheral immunoglobulin secretion. Thus, “B cell-deficient” should not be taken to mean that B cells or IgA are absent from every anatomical compartment. By contrast, the immunoglobulin secretion-deficient IgMi model also altered intestinal B–T-cell interactions and steatohepatitis [2]. These model-specific findings distinguish local B-cell-associated inflammatory activity from peripheral antibody-associated fibrogenesis; they do not establish that MASH inflammation is universally antibody-independent.

### 4.2. IgA-Associated Macrophage Responses and Fibrogenesis

The findings above suggest two separable but potentially coexisting contributions to MASH: metabolically programmed intestinal B cells can facilitate CD8+ T-cell-associated inflammation, whereas secreted antibodies can engage hepatic myeloid pathways linked to fibrogenesis (Figure 2B). In the models studied by Kotsiliti et al., loss of IgA availability or immunoglobulin secretion reduced fibrosis, and Fc receptor common γ-chain (FcRγ) deficiency also reduced fibrosis despite persistent steatohepatitis [2]. FcRγ is a shared signaling component of several receptors and does not demonstrate signaling through a specific IgA-binding receptor. Moreover, standard laboratory mice lack an ortholog of human FcαRI/CD89; murine FcRγ-dependent fibrosis must therefore not be equated with human CD89-mediated signaling. Human single-cell profiling of cirrhotic liver identified expanded TREM2+CD9+ scar-associated macrophages and inferred interactions with endothelial and collagen-producing mesenchymal cells in fibrotic niches, providing an anatomical context for macrophage–stromal crosstalk without establishing IgA-specific signaling [42].

### 4.3. The IgA-Enriched Phenotype and Clinical Progression

Clinical studies have linked serum IgA to the severity of steatotic liver disease. Earlier work associated elevated IgA with advanced fibrosis in biopsy-characterized non-alcoholic fatty liver disease, the nomenclature then used for MASLD, supporting investigation of its value beyond conventional biochemical markers [43]. Prospective outcome data establish fibrosis stage as a major determinant of liver-related complications, setting the clinical benchmark against which additional immune biomarkers should be evaluated [44]. Kimura et al. evaluated longitudinal outcomes and identified an IgA-enriched subgroup among 349 patients with MASLD. The 5-year cumulative incidence of liver-related events in this subgroup was 24.0%. Serum IgA ≥318 mg/dL was associated with subsequent events after adjustment for advanced fibrosis (hazard ratio, 3.17; 95% confidence interval, 1.27–7.91). This prognostic association was supported by an independent elastography-based cohort (n = 287) and an external multicenter cohort (n = 272) [9].

## 5. IgA-Associated Immune and Stromal Networks in Hepatocellular Carcinoma

### 5.1. IgA-Producing Cells and Impaired Tumor Surveillance

HCC highlights a functional distinction between immune responses that sustain liver injury and those that eliminate emerging malignant cells. Shalapour et al. demonstrated that IgA-producing cells accumulated in inflammation-associated hepatocarcinogenesis and suppressed protective CD8+ T-cell responses through PD-L1 and IL-10. Genetic and immunological interventions supported a causal role for these cells in suppressing tumor-directed immunity in the mouse models tested. Human tissue findings established the presence of IgA-positive cells in diseased livers and HCC but did not independently establish the same causal pathway in patients [33]. Figure 2C contrasts this mouse cellular mechanism with findings from human macrophage and patient-derived CAF functional assays.

In these mouse models, the immunoregulatory effect reflects the functions of IgA-producing cells; it does not demonstrate that secreted IgA directly inhibits T cells. Not every hepatic CD8+ T-cell response is protective. In separate models of steatohepatitis-associated HCC, Pfister et al. identified pathogenic CD8+ T-cell responses and found that PD-1 blockade could aggravate tumor development under the experimental conditions examined [45]. Restoring tumor surveillance therefore requires attention to T-cell specificity, functional state, and disease context.

### 5.2. IgA-Bound Monocytes and Macrophages

In human diseased liver and HCC tissue, our previous study found that IgA-bound monocytes and macrophages were associated with high PD-L1 expression [8]. In separate human monocyte-derived macrophage assays, IgA stimulation increased PD-L1 expression through a CD89-dependent response [8]. Importantly, tissue IgA binding associated with PD-L1 staining does not by itself demonstrate receptor engagement or an IgA-triggered functional response in the sampled tumors.

The tissue observations and functional assays provide different types of evidence. Together with the cooperation between IgA immune complexes and microbial signals discussed in Section 2.4 [31], they suggest that IgA-responsive myeloid cells may participate in both inflammatory and inhibitory programs, without establishing a causal contribution to HCC progression in patients.

Yes-associated protein (YAP) is a candidate downstream mediator: IgA promoted YAP nuclear localization, while pharmacological YAP inhibition reduced PD-L1 and cytokine transcripts [8].

However, YAP inhibition alone did not significantly reduce tumor size in the reported syngeneic experiment [8]. Thus, the macrophage signaling phenotype does not demonstrate that systemic YAP inhibition controls tumor growth.

### 5.3. IgA-Responsive Cancer-Associated Fibroblasts

In functional assays, patient-derived cancer-associated fibroblasts (CAFs) exposed to serum-derived IgA dimers increased IL-6 and CXCL12 secretion and PD-L1 expression. Coculture with CAFs alone reduced indicators of cytotoxic T-cell function, and IgA pretreatment further increased T-cell PD-1 expression [6]. The additional IgA response therefore modifies an existing stromal suppressive state; it should not be presented as the sole cause of CAF-mediated T-cell dysfunction.

CD71 knockdown reduced surface IgA binding, fibroblast activation protein (FAP) upregulation, and STAT3 phosphorylation in patient-derived CAFs [6]. These findings implicate CD71 in the response but do not separate IgA recognition from its role in transferrin-dependent iron uptake. Receptor-specific immune effects and inhibition of downstream pathways therefore require separate interpretation. Consequently, surface IgA positivity or CD71 staining in patient tissue should not be interpreted as proof of CD71-dependent functional activation without an appropriate perturbation assay.

### 5.4. Linking Chronic Liver Disease to the Tumor Microenvironment

The evidence for IgA-producing cells, IgA-bound myeloid cells, and IgA-responsive fibroblasts arises from distinct study designs: causal mouse interventions for the first population, human tissue associations and macrophage functional assays for the second, and patient-derived CAF assays for the third [6,8,33]. Their coexistence as a single spatially connected suppressive circuit has not been demonstrated in humans. HCC varies in immune composition and metabolic programs [46,47,48]; single-cell and spatial multi-omics approaches can help characterize CAF–immune interactions and distinct tumor niches [49].

IgA-associated fibrogenesis in the tested MASH mouse models provides a rationale for investigating the premalignant liver [2], but a shared IgA-driven causal progression from MASH fibrosis to HCC has not been demonstrated. The persistence of particular antibody clones across non-tumoral and tumor tissue, and any intestinal contribution to those clones, remain unresolved.

Clinical translation will also require evidence beyond tissue associations. Molecular analyses of atezolizumab–bevacizumab trials linked pre-existing antitumor immunity to treatment outcomes, illustrating the importance of the broader immune context [50]. IgA-associated features would require separate validation as treatment-selection biomarkers.

In neoadjuvant HCC immunotherapy, higher intratumoral tertiary lymphoid structure (TLS) density at surgery was associated with pathological response and longer relapse-free survival [51]. These B-cell-containing structures contrast with IgA-associated suppression: a B-cell-rich humoral environment can accompany protective antitumor immunity. In perioperative settings, post-treatment tissue correlates must be distinguished from pretreatment selection markers [52].

Table 1 compares evidence types and interpretive limits across ALD, MASLD/MASH, and HCC; these findings should not be treated as proof of a single causal gut–liver IgA pathway in patients.

## 6. Therapeutic Opportunities and Future Directions

### 6.1. Modulating Antibody Production, Trafficking, and Protective Export

Antibody production and plasma-cell maintenance are potential intervention points when a defined pathogenic antibody pool drives injury; however, this does not establish intestinal suppression as the preferred strategy. The persistence of long-lived plasma cells and the failure of rituximab to reduce galactose-deficient IgA1 in an IgA nephropathy trial illustrate why B-cell depletion may spare an established antibody pool [53,54].

Inhibition of APRIL and B-cell activating factor (BAFF) targets pathways that support humoral responses. Phase 3 trials in IgA nephropathy reported reduced proteinuria with sibeprenlimab and atacicept; sibeprenlimab also reduced circulating galactose-deficient IgA1 [55,56]. These trials demonstrate renal, not hepatic, efficacy and do not establish intestinal selectivity. Gut-directed delivery is a separate concept: targeted-release budesonide improved renal outcomes in NefIgArd, but its corticosteroid actions extend beyond IgA suppression [57].

Cellular trafficking is a distinct therapeutic target. FTY720 reduced liver injury and altered IgA-associated measures in ethanol-fed mice but broadly affects lymphocyte distribution [37]. Vedolizumab altered oral cholera vaccine responses while preserving responses to a parenteral vaccine, illustrating that anatomical targeting can still affect mucosal immunity [58]. Neither intervention establishes an IgA-specific gut-to-liver blockade. Protective export offers a different therapeutic strategy: hepatocyte pIgR restoration in deficient mice increased intestinal IgA and reduced bacterial translocation and ethanol injury [3]. In these experimental mice, enhancing protective export reduced liver injury; whether an export-directed intervention can reduce pathogenic hepatic IgA exposure or disease progression in humans remains untested. For clinical translation, export-directed interventions should demonstrate functional intestinal antibody delivery in humans, whereas trafficking-directed interventions should distinguish changes in IgA-producing cells from broader lymphocyte redistribution.

### 6.2. Targeting Hepatic IgA Effector Responses

Local effector responses provide another intervention point when pathogenic cells or antibody pools persist within the liver. Human macrophage CD89 blockade reduced IgA-induced PD-L1 expression [8]. Anti-FcαRI antibodies also prevented and treated skin inflammation in models using human IgA and human FcαRI-transgenic mice [32], consistent with the species distinction in Section 2.4. These findings support investigation of receptor-directed approaches but do not establish liver efficacy or selective effects on hepatic macrophages.

**Figure 3:**
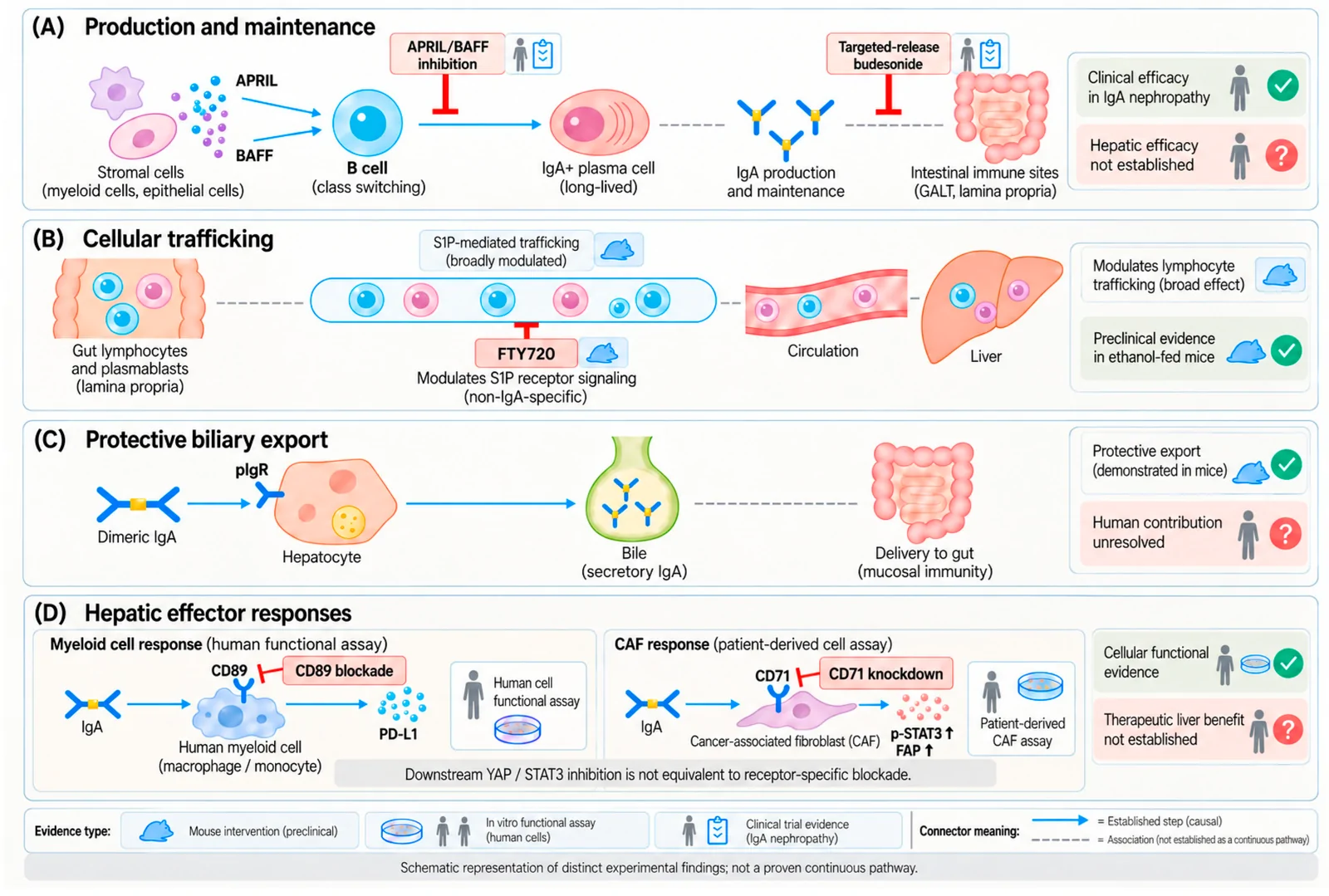
Compartment-specific therapeutic opportunities and their evidence. (**A**) Production and maintenance. APRIL/BAFF pathway inhibition and targeted-release budesonide delivery are separate approaches with clinical efficacy in IgA nephropathy, not established hepatic efficacy or IgA-specific intestinal suppression [55,56,57]. (**B**) Cellular trafficking. FTY720 reduced hepatic IgA-associated measures and ethanol-induced injury in mice through broad S1P receptor modulation; an IgA-specific trafficking blockade was not established [37]. (**C**) Protective biliary export. Hepatocyte pIgR restoration in ethanol-fed pIgR-deficient mice increased intestinal IgA and reduced bacterial translocation and liver injury [3]. A comparable protective contribution from human hepatocyte transport remains unresolved. (**D**) Hepatic effector responses. CD89 blockade reduced IgA-induced PD-L1 in human myeloid cells [8], whereas CD71 knockdown attenuated CAF activation and STAT3 phosphorylation in patient-derived fibroblasts [6]. These cellular perturbations do not establish the benefit of systemic YAP or STAT3 inhibition. Solid blue arrows denote experimentally supported directional steps within individual mechanisms, whereas gray dashed, non-directional connectors indicate conceptual associations between distinct processes without implying a proven continuous causal pathway. Red blunt-ended marks indicate intervention points. Evidence icons and labels distinguish mouse preclinical interventions, in vitro functional assays using human cells, and clinical trial evidence; the clinical trial icons in panel (A) refer specifically to IgA nephropathy rather than liver disease. These evidence types do not imply equivalent therapeutic readiness. All strategies require assessment of bacterial containment and epithelial barrier function alongside liver outcomes. Take-home message: Compartment-directed interventions are mechanistically distinct, and effects demonstrated in renal disease, mouse models, or cell assays do not by themselves establish clinical benefit in liver disease.

CD71 knockdown attenuated IgA-associated CAF activation, but selective blockade of IgA binding must be distinguished from loss of transferrin uptake [6,59]. Likewise, YAP-associated myeloid and STAT3-associated stromal responses are downstream candidates, not evidence that systemic inhibitors reproduce cell-specific receptor blockade [6,8]. Combination strategies must account for checkpoint inhibitor-associated liver and gastrointestinal toxicities [60]. Figure 3 distinguishes intervention targets and the evidence supporting each approach. Targeting PD-L1- or IL-10-associated suppression by IgA-producing cells also requires a strategy that preserves beneficial B-cell and TLS responses [33,51]. These approaches should be evaluated using cell-specific immune effects and tumor outcomes rather than antibody abundance alone.

At present, no IgA-directed therapeutic strategy has demonstrated liver disease-specific clinical efficacy in patients with ALD, MASLD/MASH, or HCC. The renal benefits reported for APRIL/BAFF-directed agents and targeted-release budesonide in IgA nephropathy cannot be extrapolated directly to hepatic disease [55,56,57]. Although hepatocyte pIgR restoration reduced ethanol-induced injury in mice, the quantitative contribution of this protective export route and the efficacy of its therapeutic restoration in humans remain unestablished [3]. Similarly, CD89 blockade and CD71 knockdown have yielded receptor-associated responses in experimental human-cell systems but have not established liver-directed clinical efficacy, cell-type selectivity in patients, or mucosal safety [6,8].

### 6.3. Biomarkers for Patient Selection and Treatment Monitoring

Biomarker development should distinguish among prognostic, treatment-selection, and target-engagement markers. Serum IgA, hepatic tissue distribution, and transcriptional programs describe distinct biological features [9]. Serum IgA has prognostic associations in MASLD but has not been validated to select patients for IgA-directed therapy [9,43].

Assay standardization should include multiplex immunofluorescence or immunohistochemistry with IgA, plasma-cell, myeloid-cell, and receptor markers, with clearly defined anatomical regions and reproducible quantification. Flow cytometry should distinguish intracellular IgA production from surface-bound IgA; IgA staining alone is insufficient to classify a cell as IgA-producing or functionally IgA-responsive [8,37]. Where feasible, functional responsiveness should be substantiated by cell stimulation or receptor perturbation.

Matched liver and serum measurements should quantify IgA1 and IgA2, monomeric and polymeric fractions, and antigen specificity or antibody repertoire where feasible. Longitudinal sampling before and after intervention, including paired tissue and blood when clinically appropriate, would help distinguish target engagement from associations with disease severity. Readouts should match the intervention: production and secretion for production-directed approaches, tracked cell distribution for trafficking, intact luminal IgA for export, and cell-specific receptor signaling for effector blockade. Reduced serum IgA alone does not demonstrate improvement in inflammation, fibrosis, or tumor outcomes.

Prospective studies should evaluate the added value of IgA-related biomarkers beyond histological fibrosis stage, the Fibrosis-4 index (FIB-4), and elastography-based liver stiffness. A multicenter MASLD study demonstrated differences in histological severity and outcomes among patients with discordant FIB-4 and liver stiffness measurements [61], providing a relevant benchmark rather than a rationale to replace fibrosis assessment with an IgA cutoff. In ALD, immune markers also need evaluation alongside established fibrosis and disease-severity measures [62]. HCC immunogenomics offers a framework for incorporating tumor genetics and immune-cell states [63], while REMARK informs prognostic reporting [64].

### 6.4. Preserving Mucosal Protection While Limiting Hepatic Pathology

All four intervention strategies must preserve effective mucosal defense. Human IgA deficiency illustrates incomplete compensation by IgM, while hepatic pIgR restoration protects ethanol-fed mice through increased intestinal delivery [3,65]. The renal benefits of atacicept [56] must also be considered alongside the increased relapse activity observed in the earlier ATAMS multiple sclerosis trial [66]. Efficacy and safety depend on the clinical indication and cannot be inferred from reductions in antibody measures alone.

### 6.5. Unresolved Questions and Priority Experiments

Four unresolved questions define the priority experiments for clinical translation:

(1) Origin of hepatic IgA responses. Compare paired intestinal, blood, non-tumoral liver, and tumor samples using B-cell receptor sequencing and spatial mapping. Shared clonotypes would support relatedness but would not establish the direction of migration.
(2) Human protective export. Quantify pIgR-associated biliary or luminal delivery, IgA molecular form, and microbial containment in matched human samples. Serum IgA concentration alone is insufficient to demonstrate functional export.
(3) Specificity of cellular trafficking. Combine lineage or repertoire evidence with selective perturbations to distinguish recruitment of IgA-related cells from general lymphocyte redistribution; no established IgA-specific inhibitor of gut-to-liver trafficking is currently available.
(4) Therapeutic efficacy and mucosal safety. Test compartment- or receptor-specific target engagement alongside independent outcomes of liver inflammation, fibrosis, or tumor control, while also assessing epithelial barrier function and infection-related safety. Human receptor-specific experiments are needed before translating murine FcRγ findings. For HCC, preclinical studies should employ mouse models that reflect the relevant underlying liver disease and experimental objectives, as no single model fully recapitulates the heterogeneity of human HCC [67].

Spatially resolved and functional studies should also determine whether IgA-producing cells, IgA-bound macrophages, and IgA-responsive CAFs coexist in connected suppressive niches or represent distinct responses. These experiments should distinguish associations in human samples from evidence obtained through causal interventions.

## 7. Conclusions

IgA links intestinal immunity to hepatic inflammation, fibrosis, and tumor immune regulation through distinct but interacting processes involving antibody production, cellular trafficking, hepatic handling, and local effector responses. Its biological effects cannot be inferred from abundance alone; rather, they depend on anatomical compartment, antibody properties, and the phenotype of responding cells. Current evidence provides a rationale for investigating disease-specific modulation of pathogenic hepatic IgA responses while preserving mucosal protection, rather than supporting indiscriminate IgA suppression. Future studies should determine when targeting antibody production or cellular trafficking, restoring protective export, or interrupting local effector pathways can improve liver outcomes without compromising mucosal defense.

## Figures and Tables

**Table 1:** Key original studies of gut–liver IgA communication.

Study	Species/Human Relevance	Evidence Type	Principal Finding	Gut–Liver Link	Interpretive Limit
Moro-Sibilot 2016 [10]	Human tissue Mouse	Mouse tracing; Human tissue observation	Peyer’s patch-derived plasmablasts populate mouse liver.	Direct mouse cell-origin evidence; commensal reactivity.	Human origin not traced.
Hendrikx 2023 [3]	Mouse	Mouse pIgR rescue intervention	Hepatocyte pIgR restoration increased intestinal IgA and reduced injury.	Functional liver-to-gut export.	Quantitative human contribution unresolved.
Kim 2026 [37]	Human Mouse	Human cell assay; mouse FTY720 intervention	Inflammatory response and conditioned-medium Huh7 apoptosis; FTY720 reduced injury.	Separate intestinal and hepatic observations; causal direction unproven.	Broad trafficking effects; cytotoxic mediators not resolved.
Ahmadi 2023 [7]	Human	Human tissue antibody assay	Cross-reactivity; tissue immunoglobulins enhanced PBMC-dependent hepatocyte killing.	Bacterial/self-antigen recognition.	Killing not assigned specifically to IgA.
Inamine 2016 [39]	Mouse	Mouse genetic deficiency	IgA loss did not reduce injury; IgM increased.	Systemic deficiency experiment.	Lifelong compensation differs from selective modulation.
Kotsiliti 2023 [2]	Human Mouse	Mouse genetics/tracing; human association	B–CD8 activation and antibody-associated fibrosis were separable.	Direct intestinal B/T-cell trafficking in mice.	FcRγ dependence does not identify CD89.
Kimura 2026 [9]	Human	Human longitudinal cohort; tissue association	IgA-enriched phenotype predicted events; portal and colonic IgA enrichment.	Human compartmental associations; EndoCAb IgG correlate.	Small colonic samples; no directional trafficking or treatment prediction.
Shalapour 2017 [33]	Human tissue Mouse	Mouse causal intervention; human observation	Cell-associated PD-L1/IL-10 impaired CD8 surveillance.	Mouse cellular causality.	Not a direct secreted-IgA effect or human origin demonstration.
Sung 2022 [8]	Human Mouse	Human macrophage functional assay; mouse intervention	CD89-dependent PD-L1; YAP-linked response.	Local IgA-bound myeloid response.	YAP inhibition alone did not significantly reduce mouse tumor size.
Park 2024 [6]	Human	Patient-derived CAF functional assay	CD71 knockdown reduced IgA binding, FAP and STAT3 responses.	Local antibody–stromal interaction.	Additional T-cell PD-1 effect differs from baseline CAF suppression; iron uptake confounds.

Evidence type describes study design and the kind of inference supported; it is not a formal certainty score or a uniform ranking of evidence. “Interpretive limit” indicates what a study cannot establish and, where relevant, the limits of extrapolation to human disease. Test material is specified in the main text where antibody exposure was manipulated. CAF, cancer-associated fibroblast; FcRγ, Fc receptor common γ-chain; PBMC, peripheral blood mononuclear cell; pIgR, polymeric immunoglobulin receptor. Disease abbreviations are defined in the text.

## Data Availability

No new datasets were generated or analyzed for this narrative review. The evidence discussed is available in the cited publications.

## Abbreviations

ALD	alcohol-associated liver disease
APRIL	a proliferation-inducing ligand
ASGPR	asialoglycoprotein receptor
BAFF	B-cell activating factor
CAF	cancer-associated fibroblast
EndoCAb	endotoxin-core antibody
FcαRI	Fcα receptor I
FcRγ	Fc receptor common γ-chain
FIB-4	Fibrosis-4 index
GALT	gut-associated lymphoid tissue
HCC	hepatocellular carcinoma
IgA	immunoglobulin A
IL	interleukin
LPS	lipopolysaccharide
MASH	metabolic dysfunction-associated steatohepatitis
MASLD	metabolic dysfunction-associated steatotic liver disease
PBMC	peripheral blood mononuclear cell
PD-L1	programmed death-ligand 1
PEDF	pigment epithelium-derived factor
pIgR	polymeric immunoglobulin receptor
S1P	sphingosine-1-phosphate
TGF-β	transforming growth factor-β
TLS	tertiary lymphoid structure
TNF-α	tumor necrosis factor-α
YAP	Yes-associated protein

## References

[1] Tilg H., Adolph T.E., Trauner M. (2022). Gut-Liver Axis: Pathophysiological Concepts and Clinical Implications. Cell Metab..

[2] Kotsiliti E., Leone V., Schuehle S., Govaere O., Li H., Wolf M.J., Horvatic H., Bierwirth S., Hundertmark J., Inverso D. (2023). Intestinal B Cells License Metabolic T-Cell Activation in NASH Microbiota/Antigen-Independently and Contribute to Fibrosis by IgA-FcR Signalling. J. Hepatol..

[3] Hendrikx T., Lang S., Rajcic D., Wang Y., McArdle S., Kim K., Mikulski Z., Schnabl B. (2023). Hepatic pIgR-Mediated Secretion of IgA Limits Bacterial Translocation and Prevents Ethanol-Induced Liver Disease in Mice. Gut.

[4] Gleeson P.J., Camara N.O.S., Launay P., Lehuen A., Monteiro R.C. (2024). Immunoglobulin A Antibodies: From Protection to Harmful Roles. Immunol. Rev..

[5] Simpson K., Baillou R., Le Roy T., Ranson A., Vazquez-Gomez M., Sterlin D., Gorochov G., Beaumont M., Clément K., Clément E. (2026). Disruption of IgA-Mediated Aggregation at Weaning Favors Mucus Encroachment by Commensal Bacteria. npj Biofilms Microbiomes.

[6] Park J.G., Roh P.R., Kang M.W., Cho S.W., Hwangbo S., Jung H.D., Kim H.U., Kim J.H., Yoo J.-S., Han J.W. (2024). Intrahepatic IgA Complex Induces Polarization of Cancer-Associated Fibroblasts to Matrix Phenotypes in the Tumor Microenvironment of HCC. Hepatology.

[7] Ahmadi A.R., Song G., Gao T.S., Ma J., Han X.M., Hu M.W., Cameron A.M., Wesson R.N., Philosophe B., Ottmann S. (2023). Discovery and Characterization of Cross-Reactive Intrahepatic Antibodies in Severe Alcoholic Hepatitis. Elife.

[8] Sung P.S., Park D.J., Roh P.R., Mun K.D., Cho S.W., Lee G.W., Jung E.S., Lee S.H., Jang J.W., Bae S.H. (2022). Intrahepatic Inflammatory IgA(+)PD-L1(High) Monocytes in Hepatocellular Carcinoma Development and Immunotherapy. J. Immunother. Cancer.

[9] Kimura T., Wakabayashi S.I., Iwadare T., Kondo S., Okumura T., Kobayashi H., Yamashita Y., Kamijo Y., Fujita K., Nakajima T. (2026). IgA-Enriched Phenotype Predicts Liver-Related Events in MASLD. J. Hepatol..

[10] Moro-Sibilot L., Blanc P., Taillardet M., Bardel E., Couillault C., Boschetti G., Traverse-Glehen A., Defrance T., Kaiserlian D., Dubois B. (2016). Mouse and Human Liver Contain Immunoglobulin A-Secreting Cells Originating from Peyer’s Patches and Directed against Intestinal Antigens. Gastroenterology.

[11] Cazac B.B., Roes J. (2000). TGF-beta Receptor Controls B Cell Responsiveness and Induction of IgA in Vivo. Immunity.

[12] He B., Xu W., Santini P.A., Polydorides A.D., Chiu A., Estrella J., Shan M., Chadburn A., Villanacci V., Plebani A. (2007). Intestinal Bacteria Trigger T Cell-Independent Immunoglobulin A(2) Class Switching by Inducing Epithelial-Cell Secretion of the Cytokine APRIL. Immunity.

[13] Reboldi A., Arnon T.I., Rodda L.B., Atakilit A., Sheppard D., Cyster J.G. (2016). IgA Production Requires B Cell Interaction with Subepithelial Dendritic Cells in Peyer’s Patches. Science.

[14] Kim M., Qie Y., Park J., Kim C.H. (2016). Gut Microbial Metabolites Fuel Host Antibody Responses. Cell Host Microbe.

[15] Mora J.R., Iwata M., Eksteen B., Song S.Y., Junt T., Senman B., Otipoby K.L., Yokota A., Takeuchi H., Ricciardi-Castagnoli P. (2006). Generation of Gut-Homing IgA-Secreting B Cells by Intestinal Dendritic Cells. Science.

[16] Inamine T., Schnabl B. (2018). Immunoglobulin A and Liver Diseases. J. Gastroenterol..

[17] Moor K., Diard M., Sellin M.E., Felmy B., Wotzka S.Y., Toska A., Bakkeren E., Arnoldini M., Bansept F., Co A.D. (2017). High-Avidity IgA Protects the Intestine by Enchaining Growing Bacteria. Nature.

[18] Landsverk O.J., Snir O., Casado R.B., Richter L., Mold J.E., Reu P., Horneland R., Paulsen V., Yaqub S., Aandahl E.M. (2017). Antibody-Secreting Plasma Cells Persist for Decades in Human Intestine. J. Exp. Med..

[19] Wilmore J.R., Gaudette B.T., Gomez Atria D., Rosenthal R.L., Reiser S.K., Meng W., Rosenfeld A.M., Prak E.T.L., Allman D. (2021). IgA Plasma Cells Are Long-Lived Residents of Gut and Bone Marrow That Express Isotype- and Tissue-Specific Gene Expression Patterns. Front. Immunol..

[20] Iversen R., Snir O., Stensland M., Kroll J.E., Steinsbo O., Korponay-Szabo I.R., Lundin K.E.A., de Souza G.A., Sollid L.M. (2017). Strong Clonal Relatedness between Serum and Gut IgA Despite Different Plasma Cell Origins. Cell Rep..

[21] Wilmore J.R., Gaudette B.T., Gomez Atria D., Hashemi T., Jones D.D., Gardner C.A., Cole S.D., Misic A.M., Beiting D.P., Allman D. (2018). Commensal Microbes Induce Serum IgA Responses That Protect against Polymicrobial Sepsis. Cell Host Microbe.

[22] Rifai A., Fadden K., Morrison S.L., Chintalacharuvu K.R. (2000). The N-Glycans Determine the Differential Blood Clearance and Hepatic Uptake of Human Immunoglobulin (Ig)A1 and IgA2 Isotypes. J. Exp. Med..

[23] Chandy K.G., Hubscher S.G., Elias E., Berg J., Khan M., Burnett D. (1983). Dual Role of the Liver in Regulating Circulating Polymeric Ig in Man: Studies on Patients with Liver Disease. Clin. Exp. Immunol..

[24] Kaetzel C.S., Robinson J.K., Chintalacharuvu K.R., Vaerman J.P., Lamm M.E. (1991). The Polymeric Immunoglobulin Receptor (Secretory Component) Mediates Transport of Immune Complexes across Epithelial Cells: A Local Defense Function for IgA. Proc. Natl. Acad. Sci. USA.

[25] Nagura H., Tsutsumi Y., Hasegawa H., Watanabe K., Nakane P.K., Brown W.R. (1983). IgA Plasma Cells in Biliary Mucosa: A Likely Source of Locally Synthesized IgA in Human Hepatic Bile. Clin. Exp. Immunol..

[26] Kim G., Chen Z., Li J., Luo J., Castro-Martinez F., Wisniewski J., Cui K., Wang Y., Sun J., Ren X. (2024). Gut-Liver Axis Calibrates Intestinal Stem Cell Fitness. Cell.

[27] Bunker J.J., Erickson S.A., Flynn T.M., Henry C., Koval J.C., Meisel M., Jabri B., Antonopoulos D.A., Wilson P.C., Bendelac A. (2017). Natural Polyreactive IgA Antibodies Coat the Intestinal Microbiota. Science.

[28] Palm N.W., de Zoete M.R., Cullen T.W., Barry N.A., Stefanowski J., Hao L., Degnan P.H., Hu J., Peter I., Zhang W. (2014). Immunoglobulin A Coating Identifies Colitogenic Bacteria in Inflammatory Bowel Disease. Cell.

[29] Li C., Cai C., Wang C., Chen X., Zhang B., Huang Z. (2025). Gut Microbiota-Mediated Gut-Liver Axis: A Breakthrough Point for Understanding and Treating Liver Cancer. Clin. Mol. Hepatol..

[30] Pasquier B., Launay P., Kanamaru Y., Moura I.C., Pfirsch S., Ruffie C., Hénin D., Benhamou M., Pretolani M., Blank U. (2005). Identification of FcαRI as an Inhibitory Receptor That Controls Inflammation: Dual Role of FcRγ ITAM. Immunity.

[31] Hansen I.S., Hoepel W., Zaat S.A.J., Baeten D.L.P., den Dunnen J. (2017). Serum IgA Immune Complexes Promote Proinflammatory Cytokine Production by Human Macrophages, Monocytes, and Kupffer Cells through FcαRI-TLR Cross-Talk. J. Immunol..

[32] Bos A., Aleyd E., van der Steen L.P.E., Winter P.J., Heemskerk N., Pouw S.M., Boon L., Musters R.J.P., Bakema J.E., Sitaru C. (2022). Anti-FcαRI Monoclonal Antibodies Resolve IgA Autoantibody-Mediated Disease. Front. Immunol..

[33] Shalapour S., Lin X.J., Bastian I.N., Brain J., Burt A.D., Aksenov A.A., Vrbanac A.F., Li W., Perkins A., Matsutani T. (2017). Inflammation-Induced IgA+ Cells Dismantle Anti-Liver Cancer Immunity. Nature.

[34] Llopis M., Cassard A.M., Wrzosek L., Boschat L., Bruneau A., Ferrere G., Puchois V., Martin J.C., Lepage P., Le Roy T. (2016). Intestinal Microbiota Contributes to Individual Susceptibility to Alcoholic Liver Disease. Gut.

[35] Leclercq S., Matamoros S., Cani P.D., Neyrinck A.M., Jamar F., Starkel P., Windey K., Tremaroli V., Bäckhed F., Verbeke K. (2014). Intestinal Permeability, Gut-Bacterial Dysbiosis, and Behavioral Markers of Alcohol-Dependence Severity. Proc. Natl. Acad. Sci. USA.

[36] Duan Y., Llorente C., Lang S., Brandl K., Chu H., Jiang L., White R.C., Clarke T.H., Nguyen K., Torralba M. (2019). Bacteriophage Targeting of Gut Bacterium Attenuates Alcoholic Liver Disease. Nature.

[37] Kim M.G., Tak K.Y., Seo D.H., Lee G.W., Cho S.W., Kwon M.H., Lee S.H., Han E.S., Ryu J.Y., Cho H.S. (2026). Blocking the Gut-Liver IgA Axis Attenuates Macrophage-Mediated Inflammation and Injury in Alcohol-Associated Liver Disease. Gut.

[38] Petrasek J., Bala S., Csak T., Lippai D., Kodys K., Menashy V., Barrieau M., Min S.-Y., Kurt-Jones E.A., Szabo G. (2012). IL-1 Receptor Antagonist Ameliorates Inflammasome-Dependent Alcoholic Steatohepatitis in Mice. J. Clin. Investig..

[39] Inamine T., Yang A.M., Wang L., Lee K.C., Llorente C., Schnabl B. (2016). Genetic Loss of Immunoglobulin A Does Not Influence Development of Alcoholic Steatohepatitis in Mice. Alcohol Clin. Exp. Res..

[40] Barrow F., Khan S., Fredrickson G., Wang H., Dietsche K., Parthiban P., Robert S., Kaiser T., Winer S., Herman A. (2021). Microbiota-Driven Activation of Intrahepatic B Cells Aggravates NASH through Innate and Adaptive Signaling. Hepatology.

[41] Bruzzi S., Sutti S., Giudici G., Burlone M.E., Ramavath N.N., Toscani A., Bozzola C., Schneider P., Morello E., Parola M. (2018). B2-Lymphocyte Responses to Oxidative Stress-Derived Antigens Contribute to the Evolution of Nonalcoholic Fatty Liver Disease (NAFLD). Free Radic. Biol. Med..

[42] Ramachandran P., Dobie R., Wilson-Kanamori J.R., Dora E.F., Henderson B.E.P., Luu N.T., Portman J.R., Matchett K.P., Brice M., Marwick J.A. (2019). Resolving the Fibrotic Niche of Human Liver Cirrhosis at Single-Cell Level. Nature.

[43] McPherson S., Henderson E., Burt A.D., Day C.P., Anstee Q.M. (2014). Serum Immunoglobulin Levels Predict Fibrosis in Patients with Non-Alcoholic Fatty Liver Disease. J. Hepatol..

[44] Sanyal A.J., Van Natta M.L., Clark J., Neuschwander-Tetri B.A., Diehl A., Dasarathy S., Loomba R., Chalasani N., Kowdley K., Hameed B. (2021). Prospective Study of Outcomes in Adults with Nonalcoholic Fatty Liver Disease. N. Engl. J. Med..

[45] Pfister D., Nunez N.G., Pinyol R., Govaere O., Pinter M., Szydlowska M., Gupta R., Qiu M., Deczkowska A., Weiner A. (2021). NASH Limits Anti-Tumour Surveillance in Immunotherapy-Treated HCC. Nature.

[46] Zhang Q., He Y., Luo N., Patel S.J., Han Y., Gao R., Modak M., Carotta S., Haslinger C., Kind D. (2019). Landscape and Dynamics of Single Immune Cells in Hepatocellular Carcinoma. Cell.

[47] Suzuki H., Mishra S., Paul S., Hoshida Y. (2025). Molecular and Immune Landscape of Hepatocellular Carcinoma for Therapeutic Development. J. Liver Cancer.

[48] Jang E.J., Sung P.S. (2026). A Novel Link between Tumor Cell Metabolism and Patient Prognosis: Editorial on “Molecular Classification of Hepatocellular Carcinoma Based on Zoned Metabolic Feature and Oncogenic Signaling Pathway”. Clin. Mol. Hepatol..

[49] Jyotishi C., Prajapati S., Patel M., Gupta R. (2026). Exploring Single-Cell and Multi-Omics Technologies and Their Role in Unraveling Tumor Heterogeneity of Hepatocellular Carcinoma. J. Liver Cancer.

[50] Zhu A.X., Abbas A.R., de Galarreta M.R., Guan Y., Lu S., Koeppen H., Zhang W., Hsu C.-H., He A.R., Ryoo B.-Y. (2022). Molecular Correlates of Clinical Response and Resistance to Atezolizumab in Combination with Bevacizumab in Advanced Hepatocellular Carcinoma. Nat. Med..

[51] Shu D.H., Ho W.J., Kagohara L.T., Girgis A., Shin S.M., Danilova L., Lee J.W., Sidiropoulos D.N., Mitchell S., Munjal K. (2024). Immunotherapy Response Induces Divergent Tertiary Lymphoid Structure Morphologies in Hepatocellular Carcinoma. Nat. Immunol..

[52] Chun H.S., Lee M., Kim T.H. (2025). Evolving Roles of Systemic Therapy in Hepatocellular Carcinoma: Neoadjuvant and Adjuvant Strategies. J. Liver Cancer.

[53] Halliley J.L., Tipton C.M., Liesveld J., Rosenberg A.F., Darce J., Gregoretti I.V., Popova L., Kaminiski D., Fucile C.F., Albizua I. (2015). Long-Lived Plasma Cells Are Contained within the CD19(-)CD38(Hi)CD138(+) Subset in Human Bone Marrow. Immunity.

[54] Lafayette R.A., Canetta P.A., Rovin B.H., Appel G.B., Novak J., Nath K.A., Sethi S., Tumlin J.A., Mehta K., Hogan M. (2017). A Randomized, Controlled Trial of Rituximab in IgA Nephropathy with Proteinuria and Renal Dysfunction. J. Am. Soc. Nephrol..

[55] Perkovic V., Trimarchi H., Tesar V., Lafayette R., Wong M.G., Barratt J., Suzuki Y., Liew A., Zhang H., Carroll K. (2026). Sibeprenlimab in IgA Nephropathy—Interim Analysis of a Phase 3 Trial. N. Engl. J. Med..

[56] Lafayette R., Barbour S.J., Brenner R.M., Campbell K.N., Doan T., Eren N., Floege J., Jha V., Kim B.S., Liew A. (2026). A Phase 3 Trial of Atacicept in Patients with IgA Nephropathy. N. Engl. J. Med..

[57] Lafayette R., Kristensen J., Stone A., Floege J., Tesar V., Trimarchi H., Zhang H., Eren N., Paliege A., Reich H.N. (2023). Efficacy and Safety of a Targeted-Release Formulation of Budesonide in Patients with Primary IgA Nephropathy (NefIgArd): 2-Year Results from a Randomised Phase 3 Trial. Lancet.

[58] Wyant T., Leach T., Sankoh S., Wang Y., Paolino J., Pasetti M.F., Feagan B.G., Parikh A. (2015). Vedolizumab Affects Antibody Responses to Immunisation Selectively in the Gastrointestinal Tract: Randomised Controlled Trial Results. Gut.

[59] Moura I.C., Centelles M.N., Arcos-Fajardo M., Malheiros D.M., Collawn J.F., Cooper M.D., Monteiro R.C. (2001). Identification of the Transferrin Receptor as a Novel Immunoglobulin (Ig)A1 Receptor and Its Enhanced Expression on Mesangial Cells in IgA Nephropathy. J. Exp. Med..

[60] Choi S.K., Woo S., Chon H.J. (2026). Immune-Related Adverse Events in Hepatocellular Carcinoma: Organ-Specific Patterns and Management Approaches. J. Liver Cancer.

[61] Rabbat J., Yang B., Lee H.W., Lin H., Tsochatzis E., Petta S., Bugianesi E., Yoneda M., Zheng M.-H., Hagström H. (2026). Histological Severity and Hepatic Outcomes in Patients with Metabolic Dysfunction-Associated Steatotic Liver Disease and Discrepant FIB-4 and Liver Stiffness Measurement. Clin. Mol. Hepatol..

[62] Rama K., Jahagirdar V., Idalsoaga F., Blaney H., Rhoads S.F., Diaz L.A., Arrese M., Arab J.P. (2026). Novel Biomarkers for Alcohol-Associated Liver Disease and Their Implications across Clinical Settings. Clin. Mol. Hepatol..

[63] Carter J.K., Cameron D.C., Villanueva A. (2026). Clinical Applications of Immunogenomics in Hepatocellular Carcinoma. Clin. Mol. Hepatol..

[64] McShane L.M., Altman D.G., Sauerbrei W., Taube S.E., Gion M., Clark G.M. (2005). Reporting Recommendations for Tumour Marker Prognostic Studies (REMARK). Br. J. Cancer.

[65] Fadlallah J., El Kafsi H., Sterlin D., Juste C., Parizot C., Dorgham K., Autaa G., Gouas D., Almeida M., Lepage P. (2018). Microbial Ecology Perturbation in Human IgA Deficiency. Sci. Transl. Med..

[66] Kappos L., Hartung H.P., Freedman M.S., Boyko A., Radu E.W., Mikol D.D., Lamarine M., Hyvert Y., Freudensprung U., Plitz T. (2014). Atacicept in Multiple Sclerosis (ATAMS): A Randomised, Placebo-Controlled, Double-Blind, Phase 2 Trial. Lancet Neurol..

[67] Kim G.Y., Kim D., Jeon J., Seo W., Park S.H. (2025). Navigating Liver Cancer with Mouse Models: A Comprehensive Overview of HCC Experimental Systems. J. Liver Cancer.

